# Some Observations on Tuberculous Disease of the Hip-Joint in Childhood and Youth

**Published:** 1904-09

**Authors:** Charles A. Morton

**Affiliations:** Professor of Surgery in University College, Bristol; Surgeon to the Bristol General Hospital and the Bristol Royal Hospital for Sick Children and Women


### SOME OBSERVATIONS ON TUBERCULOUS DISEASE OF THE HIP-JOINT IN CHILDHOOD AND YOUTH,

### WITH SPECIAL REFERENCE TO EXCISION OF THE JOINT.

BY

CHARLES A. MORTON, F.R.C.S.,

*Professor of Surgery in University College, Bristol;*
*Surgeon to the Bristol General Hospital and the Bristol Royal Hospital for Sick Children and Women.*

---

As during an experience of eight years as surgeon to the Children's Hospital I have seen many cases of tuberculous disease of the hip, and have excised the joint for this disease in twenty-four cases[1] under the age of twenty years, I propose, in this paper, to discuss the question of operative treatment, and to refer to other matters in connection with the disease.

Taking first the question of operative treatment in hip disease, I may say that I believe the removal of the head of the femur and diseased foci in the acetabulum (not merely of definite sequestra) reduces the length of the period of recovery very considerably, and may in some cases turn the scale in favour of recovery rather than of persistent suppuration. I have had no death from the operation in a series of twenty-six cases,[2] so that I do not regard the risk as great. No doubt a large number of patients will recover if the abscess is simply opened and drained aseptically, but they will require drainage for a much longer time than if the diseased bone, and as much of the diseased soft parts as possible, are removed at the same time. I regard the indication for excision to be the presence of suppuration in and outside the joint, and although I believe the removal of the diseased head, &c., may do good in cases in which septic disease is

[1] I have included one case in the twenty-four which was not truly an excision, as the whole head was not removed; but as a large sequestrum was removed from the head, it seems desirable to include it.

[2] The only deaths were: (1) Tubercular meningitis two months after operation, and *post-mortem* disease in the hip was found replaced by fibrous union; (2) pulmonary tuberculosis and pneumothorax three months after operation.

present, it is in cases of unopened abscesses that I have had the most satisfactory results, the discharge ceasing, and the disease coming to an end, much more rapidly than would, I think, have occurred with simple drainage of the abscess. In these cases of excision the range of movement in what remains of the joint is often surprisingly free, and I have notes of cases in which the thigh could be flexed to a right angle, and abduction and adduction be carried out to a wide range. Excision of the head may leave rather more shortening than recovery without; but in these suppurating cases there is likely to be considerable wearing away of the surface of the head, and very likely wandering of the head upwards from erosion of the posterior lip of the acetabulum, and thus shortening is produced. Moreover, it is in the lower end rather than the upper end of the femur that growth chiefly takes place. In some cases, however, after excision suppuration does not cease; but this is much less likely to occur after excision than after simply dealing with the abscess sac. I have only once seen the wound inoculated with tubercle, and this was the scar tissue (and surrounding muscles) after previous drainage of the abscess, when at the second operation I excised the joint. At the first operation the abscess, which communicated freely with the joint, was opened, flushed and scraped out; but the only disease found in the head of the femur or the acetabulum consisted in two minute (pin-point) pits in the cartilage of the femoral head, but much soft tuberculous tissue was present in the synovial membrane. The ligamentum teres had disappeared, and the joint surfaces were separated by an effusion of pus into the joint, so that both the head of the femur and the acetabulum could be thoroughly explored. The discharge very rapidly ceased; but another abscess formed in a few months, and in the second operation the scar tissue of the joint and the surrounding muscles were found infiltrated with caseous foci. At this time the cartilage over the femur was thin and blue in parts, but only an eroded portion of the acetabulum had to be gouged away. I have not included this case amongst my cases of excision. A sinus remained for some time after the second operation, and I do not know if it has yet healed. In two or three of my cases of excision I have seen the scar tissue break down, and then finally heal up again. This may possibly have been due to an infection.

Almost all surgeons in this country are agreed that when abscess forms in hip disease it should be freely opened and flushed and scraped out; but many think that this is enough, and that excision of the joint is not required, whilst others believe that it is better to do the more complete operation. As I have already stated, my own opinion is that much time in recovery is saved by excision. The sequestra we often meet with would certainly take a long time to come away if we did not remove them, and after sawing off the eroded head, gouging out caseous foci in the acetabulum, and cutting and scraping away all diseased soft parts, we frequently get rapid subsidence of suppuration and early healing. Moreover, a pelvic abscess communicating with the joint through a perforated acetabulum is well drained by excision of the head; and such a condition is not very rare, though, as I shall presently show, my own experience does not indicate that perforation of the acetabulum is as common as some writers maintain. But sequestra in the joint I have found to be quite common, for I have looked through my records of these twenty-four cases, and I find that they were present in no less than ten, and in most of the cases several were present either in the head or acetabulum, most often in the latter. One sequestrum measured one inch by half an inch.

I am no advocate of excision in an early stage of hip disease before there is evidence of suppuration in the joint. I think such cases have a very good chance of recovery with rest to the joint; and I do not believe (as some surgeons maintain) that we should operate to remove tuberculous tissue with a view to prevent general infection. We cannot be certain that we remove all such tissue. Who would think of doing an arthrectomy for diffuse sarcoma of a joint? Cutting through tuberculous tissue may just open up the path of the very infection we aim to avoid. Several surgeons who only flush and scrape out hip abscesses, and do not deal with the diseased bone in the joint, are strongly in favour of arthrectomy in suppurating tuberculous disease of the knee joint, and there is no doubt we have far better opportunity of cutting away all the tuberculous soft tissue in this joint than we can possibly have in the hip. Nevertheless, I believe that we can scrape away much pulpy tissue from the hip and even cut away a fair amount of tissue.[1] I always have operated by the anterior incision, between the sartorius and the tensor fasciæ femoris, and deeper down, between the rectus and gluteus minimus. No muscle is divided, and if arterial branches are cut they are only of the external circumflex artery, and the external cutaneous nerve if exposed can readily be drawn aside. It is sometimes difficult to get the detached head out through this incision, and it is often useful to employ a lithotomy scoop to lever out the head. I always have one ready in cases of hip excision. That the anterior incision is in a bad position for drainage matters little, unless septic sinuses are present, and then a counter opening behind the trochanter can readily be made, guided by the finger within the joint. The question of drainage in these cases of hip excision is an important one. I have drained all my cases. Provided that the surgeon can dress the case himself, or can get the dressing done by someone in whom he has complete confidence as to antiseptic details, I believe that drainage is the best plan. The presence of the tube is harmless, and so long as pus is formed it can flow away, and thus no reopening of an abscess will become necessary. If, however, the surgeon cannot feel confident that septic infection will not take place at the dressing, or if the opening has to be made near the anus or urethra (rarely so in hip disease), then I think it is better to dispense with a drainage-tube and take the risk of re-collection of pus.

In a recent paper on hip diseases[2] three writers are quoted as giving the percentage of cases of hip disease in which there is perforation of the acetabulum as a very large one. Ménard states that in 235 cases it occurred in 100; Vincent, of Lyons,

[1] Since this paper was written I have cut away a mass of gelatinous tuberculous tissue the size of a hen's egg in excising the hip-joint in an adult.

[2] *Practitioner*, 1903, lxx. 89.

reports 12 in 52 cases; and Ollier and Haberern say it is present in one-third of all bad cases. It has been only twice present in my own series of 26 cases, and yet they had all advanced to abscess formation outside the joint.

In one of my cases I had to excise both hip joints. This was a boy, aged 6, in the Children's Hospital. The left hip was operated on in December, 1897, and when seen a year after all movements in the joint were fairly free. Three months later he was re-admitted with signs of disease in the right hip, and again admitted in October, 1899, with abscess in connection with it, and this joint was excised in November, 1899; the wound was soundly healed a month later. I have also excised the hip joint on one side in another case of bilateral disease, and the contrast between the conditions on the two sides is very striking. The child, aged 6, came under my care in the Children's Hospital in November last with septic sinus associated with hip disease on the left side. An abscess in connection with the joint had been opened in another hospital a year before. Whilst she was in the Children's Hospital signs of disease were recognised in connection with the right hip, and in February of this year an abscess formed there and I excised the right hip, and in March the wound was healed, though subsequently a little excoriation appeared on the top of the scar.

An important point bearing somewhat on the treatment of hip disease is the part of the femur most affected, or primarily affected. The general statement in the books at the present time is that the disease starts in the interior of the head or neck, some say in one spot, some in another, but the majority agree that it is in the interior of the bone, and Erichsen gives the record of one case which supports this view. In several of my cases of excision there has been no evidence of disease in the interior of the head or neck, but only surface erosion such as it is agreed is the usual condition in the tuberculous disease of the knee joint. In other cases there have been soft, purulent foci in the head and neck, but it is not possible to say whether this is primary or secondary to a surface erosion, always present, and which itself is very likely secondary to disease starting in the soft tissues of the joint. Because such surface erosion is not rarely met with without affection of the interior of the bone, and that it is always present even when there is such affection, I should be inclined rather to regard it as the primary condition. Moreover, I have records of three cases in which clearly the disease started in the soft tissue of the joint and just under the periosteum of the neck of the femur. It is generally admitted that tuberculous disease of the knee joint starts most often in the synovial membrane, and if it starts there most frequently in this joint, probably it does so in all joints, for there is no reason why it should start in the interior of the bone most frequently in one joint and in the synovial membrane in another.

But even if the disease only involves the surface of the head, we must nevertheless excise the whole head, for we cannot remove the eroded surface and inspect the cut surface left to see if the bone is sound in the hip, as we can in the case of the knee joint. Nor can we as a rule reach the disease in the acetabulum without excision of the head of the femur. I will now record three of the cases which seemed to me to be examples of disease starting in the soft tissues of the joint. I do not of course maintain that three cases can settle the question as to the structure in which the disease usually arises, but it is a significant fact that in all three the disease seemed to start in the soft structure of the joint, or just under the periosteum, rather than in the interior of the bone.

A child was in the Children's Hospital with hip disease of six weeks' duration and died from pericarditis. Tubercles were found in the lungs. The synovial membrane of the hip joint was abnormally vascular, and much thickened. The periosteum of the neck was at one spot separated from the bone by a small quantity of pulpy tissue, and the surface of the bone was slightly eroded beneath. The ligamentum teres was softened, and gave way in the removal of the head from the acetabulum. There was only one small erosion of the articular cartilage, and that was in the acetabulum. A section of the head and neck of the femur showed no disease in the interior of the bone.

The second case was, I think, the earliest I have had of tuberculous disease of the hip. The disease was present in an infant 13 months of age, and had existed for six months, death occurring from tubercular disease of the brain. Pus was present in the hip joint, and there was tuberculous disease of the soft tissues of the joint. The ligamentum teres was destroyed. The only morbid change in the cartilage or bones was some little pits in the cartilage.

The third case I have already referred to as one on which I operated a second time for recurrent abscess. Although in this case no section of bones was made, yet it seems to me highly probable that the disease started in the synovial membrane.

I have often found that though the articular surfaces may be extensively eroded, yet no grating can be made out in the joint on free movement under the anæsthetic. There are two explanations given of this: one is that the eroded surfaces are covered by granulations and thus rendered smooth; and the other is that a piece of detached cartilage gets between them. I have, however, found extensive surface erosion, with absence of grating, and neither condition present. The explanation is, I believe, that the eroded surfaces are very moist, and hence not really rough enough to grate. Although adduction with flexion is the usual position in the late stage of the disease, I have several times seen cases of marked abduction even with external abscess.

How extremely common the presence of even a large abscess connected with joint disease without any pyrexia, morning or evening, is hardly sufficiently recognised.

Fixation of the joint, during the active stage of hip disease, is of course due to the contraction of the surrounding muscles, and yet again and again when the patient is deeply under an anæsthetic I have found that such contraction does not subside, and some flexion or adduction persists.

Therefore when we fail to rectify position by weight extension in hip disease, we must not expect that the administration of an anæsthetic will always remove the remaining deformity. It will probably considerably reduce it, but even then weight extension may have to be continued for some time to arrest what deformity is left, for forcible rectification would impose too great a strain on the joint, and might light up into greater activity the disease there present.

There is one condition which in my experience is very common in tuberculous hip disease, though very little is said about it in books, and that is the enlargement of some of the deep iliac glands, just above Poupert's ligament—not inguinal glands, but deep iliac glands lying on the vessels. I have several times noticed that a condition of marked thickening about the joint, especially if associated with definite œdema, is soon followed by signs of abscess. I should very carefully watch all such cases for evidence of suppuration. And this leads me to draw attention to the need for systematic inspection of all hip cases at regular intervals—certainly every month—for the parents fail to recognise abscess formation, because there is not necessarily any increase of pain, and even the doctor occasionally, if he finds a normal temperature, is apt to think that a swelling forming near the joint cannot contain pus. It is well also to remember that in some of these cases the fluid removed by an exploring syringe looks like turbid serum rather than pus, and yet when the abscess is opened abundant caseous débris and purulent fluid are found within the abscess sac. I should like to call attention to the danger there is in the exploration of doubtful swellings, which may be abscesses, in connection with hip disease, unless the greatest care is taken with the sterilisation of skin and instruments, and unless an effective antiseptic dressing be prepared for application in case of leakage from the puncture. For the surgeon's chances of success with drainage or excision are very seriously reduced by the introduction of any septic organisms. And although it does not apply specially to hip disease, I would like to point out that a definite loose sequestrum removed from the acetabulum or head of the femur in tuberculous disease of the joint, may present on section so many little soft red spots, that if it had been still attached to any living structure, it would probably have been regarded as itself still living. Probably in this way we may now and again leave dead bone, which we regard as simply bare bone, as in dealing with caries of the rib.

There is one fact which fortunately is very striking in the treatment of hip disease, that is the great relief to pain which the application of weight extension gives. It does not, of course, pull the joint surfaces apart; it simply prevents reflex contraction of the muscles, pressing them together, and it should be so applied as not to increase interosseous pressure by leverage, *i.e.* it should be in the line of deformity.

In Treves's book on surgical anatomy is related the case of a child who was admitted into hospital with a splint on the knee joint, but was suffering from hip disease with only referred pain in the knee. I have had a case of hip disease sent into hospital in which incision had been made into the neighbourhood of the knee joint for the relief of pain due to disease of the hip joint, which I subsequently had to excise.

